# And Baby Makes Three: Genomic Imprinting in Plant Embryos

**DOI:** 10.1371/journal.pgen.1003981

**Published:** 2013-12-05

**Authors:** Hugh Dickinson, Stefan Scholten

**Affiliations:** 1 Department of Plant Sciences, University of Oxford, Oxford, United Kingdom; 2 Biocenter Klein Flottbek, Developmental Biology and Biotechnology, University of Hamburg, Hamburg, Germany; University of Minnesota, United States of America

Genomic imprinting results in the preferential expression of alleles after fertilization, depending on their parent of origin. Imprinting is epigenetically controlled and has evolved independently in plants and animals [1]. This striking convergence, combined with a degree of conservation within kingdoms, strongly suggests that imprinting confers a significant fitness advantage. Current thinking holds that this advantage lies in the ability to control resource flow from mother to offspring. The drivers for selection are less clear but may include parental conflict [2], [3], adaptive integration of offspring and maternal genomes [4], or a combination of the two.

Data on imprinted gene function and expression pattern from mammals [5] support these ideas, and there is also a growing body of similar evidence from plants. For example, the parental dosage-related enhanced or repressed seed development that can follow interploidy crosses has been proposed to be mediated by disruption of allele-specific genomic imprinting [6]–[8]. There is also evidence from maize that parental dosage regulates genes controlling key stages of seed development [9], and, in *Arabidopsis*, imprinted Polycomb Group (PcG) proteins [10] have been shown indirectly to control seed size. Most recently, an unambiguous role for imprinting control of nutrient transfer was demonstrated using the imprinted *meg1* gene in maize, which directly regulates maternal provisioning of the embryo and controls ultimate seed composition and size in a strictly gene dosage–dependent manner [11]. This clear involvement of imprinting in quantitative aspects of seed development has obvious implications for agricultural traits, and imprinted genes may represent an unrecognized pool of variation that can be exploited for crop improvement.

**Figure 1:**
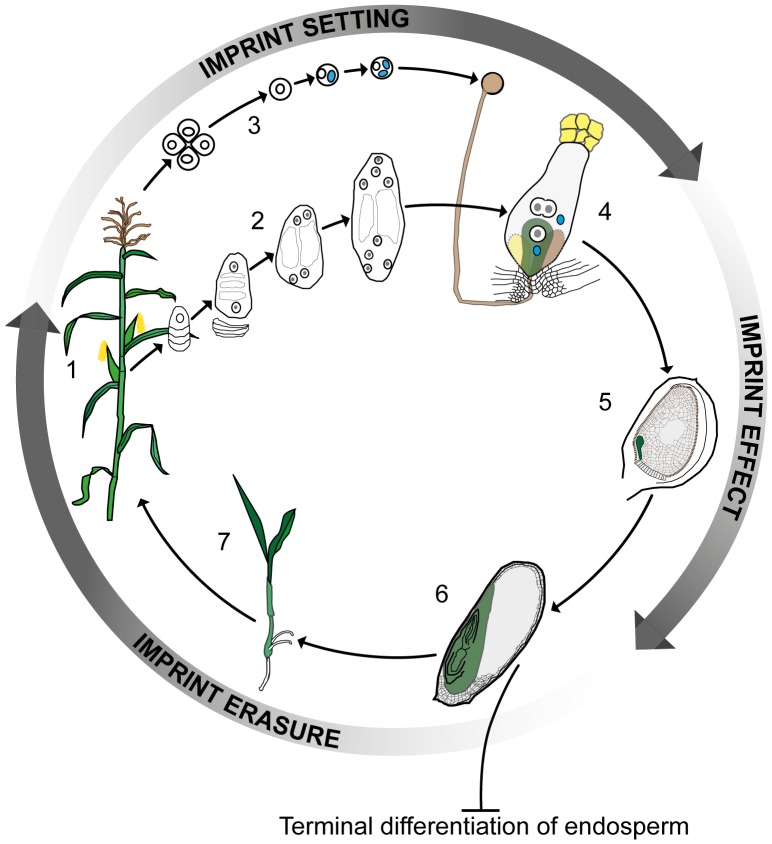
Imprinting, double fertilization, and the plant life history. A plant at flowering (1) develops female and male reproductive organs from somatic tissue in which spore mother cells undergo meiosis and undergo a haploid phase of mega- (2) and micro- (3) gametophyte development. The megagametophyte, or embryo sac, forms two female gametes over the course of three mitotic divisions—the central cell and the egg cell—in addition to two synergid cells that assist in the fertilization process. The microgametophyte, or pollen grain, undergoes two mitotic divisions to form a vegetative cell and two male gametes, the sperm cells (blue). At fertilization (4) the pollen tube delivers the two sperm cells to the receptive synergid cell. One sperm cell fertilizes the egg cell (green) and the other sperm cell nucleus combines with the two polar nuclei of the large central cell of the embryo sac. Within the ovule (5) the fertilized central cell subsequently develops into the endosperm (grey), and the fertilized egg cell forms the embryo (green). In the seed at maturity (6), the endosperm is terminally differentiated and does not contribute genetically to the next generation, whereas the embryo germinates (7) and develops into the mature plant. Genomic imprinting in the embryo requires erasure and resetting of imprinting marks according to the sex of the gametes. Clearly, parental imprints need to be set during gametophyte development and to regulate allelic gene expression during early embryo and endosperm development. Currently available data indirectly indicate that the erasure of imprints occurs during late embryo development or germination. ***Color key:*** generative cell, sperm cell, and sperm cell nuclei: blue; central and egg cell nuclei: white with grey nucleolus; pollen grain, pollen tube, and receptive synergid: brown; egg cell, embryo, seedling, and plant: green; central cell and endosperm: grey.

Plants and mammals have been reported to differ significantly in how imprinting affects the products of fertilization. While both kingdoms have evolved extra-embryonic organs to aid the process of nutrient flow from the mother to the offspring—the placenta in animals and the endosperm in plants—the placenta forms an integral part of the embryo, whereas the endosperm results from the fusion of a second sperm with a diploid accessory cell to the egg. The endosperm thus develops as a triploid structure that makes no genetic contribution to the next generation. In animals, imprinting regulates gene expression both in embryos and in other tissues during subsequent development. However, the majority of the data from plants to date has pointed to imprinting being restricted to the endosperm. Thus, while embryonic imprinting in mammals requires active reprogramming to erase and reset parent-specific imprinting marks according to the sex of the germline, it has been believed that this was not the case for plants, the endosperm being terminally differentiated (Figure 1).

This view of the endosperm as the sole site of imprinting in plants was first challenged by data from maize, which showed the *mee1* gene to be imprinted in the embryo [12]. More recent genome-wide approaches involving RNA sequencing (RNA-seq) of reciprocal crosses in *Arabidopsis* [13], rice [14], and maize [15] have also indicated the presence of several potentially imprinted genes in embryos. The study of imprinting events in plant embryos is complicated by their location deep in maternal tissue in close association with the endosperm and other tissues. This is particularly the case for *Arabidopsis*, where the embryo and its progenitor cells are extremely small. Despite these difficulties, in this issue of *PLOS Genetics* Raissig et al. [16] describe how they have risen to this challenge and, based on microarray data from gametes [17], [18] and RNA-seq results from early *Arabidopsis* embryos after unilateral crosses between two different ecotypes [19], identified a set of transcripts with clear hallmarks of imprinting. These transcripts are expressed de novo after fertilization and derived from only one parental allele. Using reverse transcription polymerase chain reaction (RT-PCR) product sequencing across polymorphisms, the parent-of-origin–dependent expression of several of the maternally expressed genes, and one paternally expressed gene, was validated in embryos generated from two sets of reciprocal crosses. Raissig et al. [16] also monitored allele-specific expression of promoter β-glucuronidase (GUS) fusion constructs for several of the maternally expressed genes following reciprocal crosses, obtaining data mirroring that from maize, where short imprinting control elements are located in the upstream promoter regions [20], [21]. Imprinting in plants thus differs strikingly from that in animals, where expression of clustered imprinted genes is regulated by long-range signals (generally noncoding RNAs) and chromosomal context is essential.

These new findings extend imprinting of embryo-expressed genes into the model species *Arabidopsis*, demonstrating that it occurs in both mono- and dicotyledonous species. Furthermore, the number of imprinted genes confirmed by Raissig et al. [16] reveals that imprinting in plant embryos is not restricted to rare events and is thus most likely to be significant for seed development. Importantly, this discovery permits the extensive genetic and “omic” resources available in *Arabidopsis* to be focused on not only the establishment of imprinting marks regulating monoallelic expression in the embryo but also on the resetting events that must take place as the embryonic tissue develops.

The epigenetic reprogramming events by which sex-specific marks are established in gametes and erased post-fertilization are complex and are only now starting to be understood. To date, two distinct, but in some ways interdependent, mechanisms of gene expression regulation have been shown to be involved in plant imprinting: DNA methylation and Histone 3 Lysine 27 tri-methylation (H3K27me3) by PcG complexes [22]. Rather unexpectedly, initial genetic analyses of a subset of the genes tested by Raissig et al. [16] indicate that the latter mechanism is involved in regulation of embryonic imprinting—although the actual parental imprinting marks in plant gametes have yet to be determined. Parent-specific DNA methylation patterning has long been recognized as the primary epigenetic mark involved in genomic imprinting and certainly fulfills this role in regulating imprinting in endosperm [21], [23]. However, the pattern of methylation in female gametes of maize [12], [21] also suggests that DNA methylation is unlikely to operate as a primary imprinting mark in embryos.

All the embryonically imprinted genes reported by Raissig et al. [16] showed bi-allelic expression in seedlings. This mirrors the situation in rice [14] and is consistent with the expression pattern of the imprinted *mee1* gene of maize, where the active allele is transitorily demethylated in the embryo [12]. These data, and the observation that DNA methylation is highly dynamic during embryo development [24], reinforce the view that imprinting in plants acts only in the developing seed and point to erasure of imprinting marks during late embryogenesis or early seed germination. This is in striking contrast to mammals, in which imprinting is reset in the germline but, in the soma, controls development after birth by influencing resource allocation, for example, through suckling, and during early care through social interactions and kin recognition [5]. Imprinting in plants may thus be restricted to seed development simply because it is the only developmental phase where offspring are maternally dependent. These exciting discoveries do, however, serve to highlight our comparative ignorance of the nature of the epigenetic marks carried by the gametic genomes and the molecular mechanisms by which they are applied and—in the embryonic tissue—erased.

The demonstration by Raissig et al. [16] that embryonic imprinting occurs in the two major plant groupings raises a number of important questions relating to the extent of imprinting in plants, the degree of its conservation between species, and its genotype dependency. However, before we can assess the importance of imprinting in seed development and plant evolution and, of course, its applicability as a tool for plant improvement, we need to know far more about the functions of the genes affected.
